# Emerging Topics in Joint Radio-Based Positioning, Sensing, and Communications

**DOI:** 10.3390/s25030948

**Published:** 2025-02-05

**Authors:** Elena Simona Lohan

**Affiliations:** Tampere Wireless Research Center, Electrical Engineering Unit, Tampere University, 33720 Tampere, Finland; elena-simona.lohan@tuni.fi

**Keywords:** wireless communications, radar, radio signals, positioning, navigation, tracking, wireless sensing, integrated sensing and communications (ISAC), radio-based simultaneous localization and mapping (radio SLAM), joint positioning, sensing, and communications (JPSAC)

## Abstract

This is an editorial paper focusing on emerging topics in joint wireless positioning, sensing, and communications. After introducing the sometimes-confusing and non-unified terminology in the field and defining the overall research area under the comprehensive terminology of Joint positioning, sensing, and communications (JPSAC), a brief state-of-the art overview is given, followed by a detailed list of emerging topics and open research questions. The ongoing Horizon Europe projects are also reviewed in relation to the emerging JPSAC. Some of the main trends in the JPSAC-related areas are related to extremely large-scale antennas and apertures, reconfigurable intelligent surfaces, the integration of terrestrial and satellite-based communication, sensing, and positioning functionalities, and cell-free or distributed networks with JPSAC functions.

## 1. Introduction and Definitions

There is a continuously increasing demand for more efficient, cost-effective, and high-performance wireless systems with wide areas of application, including, for example, telecommunications, maritime, air and land transportation, environmental monitoring, e-health, defense, and industrial applications. By integrating or offering joint solutions for wireless positioning, sensing, and communications, one can unlock new possibilities and drive innovation in multiple fields and businesses. **Wireless positioning** refers to the process of determining the location of a device or object using wireless or Radio Frequency (RF) or, simply, radio signals. This concept is closely related to the concepts of wireless tracking (i.e., monitoring and tracking the location and movement of objects, people, or animals in time) and navigation (i.e., determining or guiding the movement of a device, person, or animal from one location to another). **Wireless sensing** refers typically to two aspects: determining the presence of an object, person, or animal in a certain space–time dimension and locating or mapping the surroundings (objects, devices, cars, persons, etc.) by employing radio signals. When the wireless sensing focuses on detecting the distances to surrounding objects and their velocities, it is also known as radar technology. **Wireless communications** refer to the transmission of data or information between devices through RF signals, without the use of physical connections such as wires or cables. Both cellular systems such as Fourth generation of cellular networks (4G), Fifth generation of cellular networks (5G), or Sixth generation of cellular networks (6G) and non-cellular systems (e.g., satellite-based communications, Internet of Things (IoT)-based communications, WiFi, etc.) are covered by the broad terminology of wireless or radio-based communications.

Traditionally, these three areas (positioning, sensing, and communications) have been addressed separately by different research communities, such as the navigation community, the radar or sensing community, and the wireless communication community. For example, the *navigation community* has focused on various location technologies to support the indoor, outdoor, and seamless outdoor-to-indoor positioning, including both RF and non-RF signals, such as Global Navigation Satellite Systems (GNSS), [1,2], Low Earth Orbit (LEO), Positioning, navigation, and Timing (PNT) [3,4], cellular 4G/5G/6G signals [5,6,7], inertial sensors [8,9], magnetic signals [10], vision or sound signals [8,11], etc.; comprehensive surveys on wireless positioning from the past five years can be found, for example, in [12,13,14]. The *radar or sensing community* has been traditionally focused on techniques for improving the detection, estimation, and classification of targets, with applications mainly targeting vehicular and autonomous systems, remote sensing, environmental monitoring, and surveillance, as well as for collision avoidance. Recent surveys in the field of wireless sensing can be found in [15,16,17]. In a broader interpretation, wireless positioning can be seen as a part of the term wireless sensing, wherein the sensing terminology refers to sensing and locating everything in the environment, including the devices of interest. Last but not least, the *wireless communication community* has focused on very broad areas, encompassing terrestrial (underwater, air, maritime, railroad, …) and non-terrestrial/space communications and covering everything communications-related from the physical layer to application layers and from model-based statistical approaches to Machine Learning (ML)/Artificial Intelligence (AI)-based approaches. A few examples of surveys pertinent to top research topics involving wireless communication over the past five years are: Ref. [18] (virtualization in future wireless communications), Ref. [19] (ML/federated learning use in future wireless communications), Ref. [20] (Reconfigurable Intelligent Surfaces (RIS) advances in THz communications), Ref. [21] (a focus on future space communications), Ref. [22] (holographic communication), Ref. [23] (a quantum computing paradigm in the context of wireless communications), etc.

In recent years, however, these three communities have started to converge and address the positioning, sensing, and communication aspects in a joint manner, driven both by the standardization efforts in Third-Generation Partnership Project (3GPP) towards increased spectral, cost, and hardware efficiency, and by the recent advancements in technologies such as ML/AI and RIS, which are enabling factors for Integrated Sensing and Communications (ISAC) and Simultaneous Localization and Mapping (SLAM) developments.

**Figure 1:**
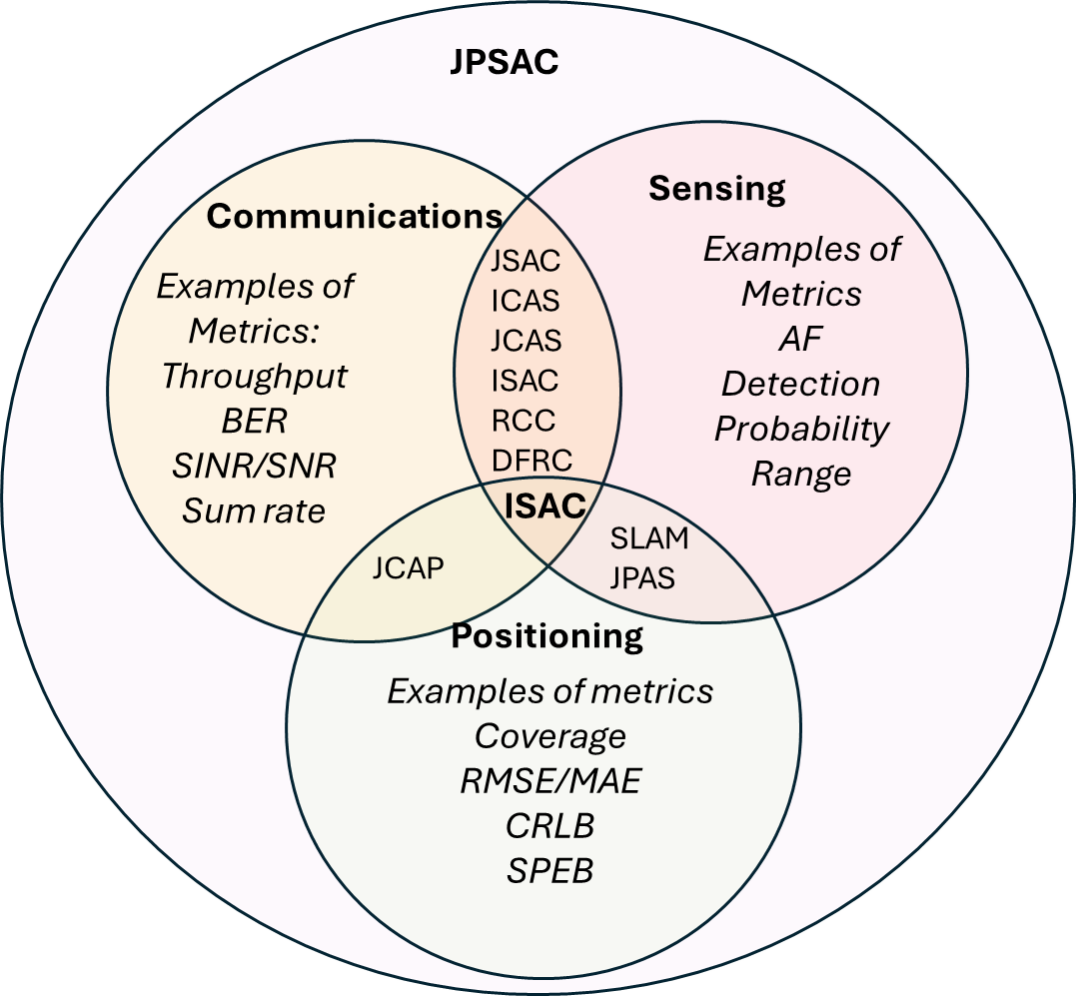
A Venn diagram of the three converging areas and existing terminology, also including the new JPSAC term; all abbreviations are explained later on in this paper and can be found in the list of abbreviations.

In this editorial, we adopt the broader term of **JPSAC** as an umbrella of all possible combinations of joining two or more of the receiver functionalities related to the positioning, tracking, navigation, sensing, radar, and communications. Thus, JPSAC will be the broader umbrella term including ISAC/Joint Sensing and Communications (JSAC) and SLAM/Joint Positioning and Sensing (JPAS), as well as the Joint Communications and Positioning (JCAP) concepts. For a straightforward illustration of how the three different areas of communications, sensing, and positioning have been addressed in a joint manner so far, we refer to the Venn diagram in Figure 1. It is to be noted that ISAC terminology has been used quite loosely in the literature so far, to describe research on both joint communications and sensing and the joint positioning, communication, and sensing.

The term ISAC, also known in the literature as JSAC, or, more seldomly, as Integrated Communications and Sensing (ICAS), Joint Communications and Sensing (JCAS), Radar Communications Coexistence (RCC), or Dual-Functional Radar Communications (DFRC), first appeared in the literature around four years ago; a few of the first detailed references to it can be found in [24] (in 2021, discussing ISAC requirements for future 6G systems), Ref. [25] (in 2021, addressing ISAC in the context of Orthogonal Time Frequency Space (OTFS) systems), Ref. [26] (also in 2021, addressing signal-processing solutions for ISAC, which they called Joint Communications and Radar sensing (JCR) in their paper), Ref. [27] (a 2022 study offering a comprehensive survey on JCAS), and [28] (addressing the RIS-assisted JCAS concept in 2022). The authors in [24] defined four categories of ISAC, namely high-accuracy localization and tracking, SLAM, augmented human senses, such as hidden object detection, and gesture and activity recognition, making their interpretation of the ISAC concept equivalent to the definitions of joint positioning, sensing, and communications rather than to the definition of joint sensing and communication, which is more popular in research papers. In general, the main goals of the ISAC involve optimizing the performance of systems that combine sensing and communication functionalities (and sometimes, also positioning functionalities); typically, the optimization of sensing and communication functions is carried out in a joint manner. The ISAC problem is often formulated as an optimization problem [29], when a communication, sensing, or joint sensing–communication metric $F_{JPSAC}\left(\cdot \right)$ is maximized (or minimized) under certain constraints (e.g., in terms of the maximum allowed transmission power, the minimum desired quality of services, etc.); the optimization is carried out after various design parameters are used, such as the signal modulation order; the number of antenna elements at the transmitter, receiver, or both; the beamforming strategy, the number of sub-carriers depending on Orthogonal Frequency Division Multiplexing (OFDM) or OTFS modulations, etc. The main tradeoffs between simultaneously achieving good sensing and communication metrics are derived from the intrinsic differences between communication and sensing/radar signals [26]: for example, communication signals typically include both pilots (unmodulated data sequences) and modulated data signals (carrying the user information), while radar signals are traditionally unmodulated signals; most communication systems nowadays rely on packet-based/discontinuous transmission, while continuous sensing requires continuous transmissions; front-end communication receivers need low noise figures, while the radar receivers have more relaxed noise–figure requirements, etc.

The SLAM terminology, which basically refers to joint positioning of targets and the sensing and positioning of the environment (walls, scatterers, other objects, persons, cars, etc.) is much older than ISAC terminology and was first used in robotics research about four decades ago. One of the first comprehensive tutorials about SLAM, also addressing a bit of the history of SLAM, is the Durrant-Whyte and Bailey tutorial [30] from 2006. At that time, the focus was fully on mobile robots equipped with various sensors, including cameras/vision sensors. The concept of radio SLAM (or radio-based SLAM) is much more recent (first appearing only a couple of years ago) and refers to SLAM methods relying purely on radio signals (without the need for visual sensors). A few of the top recent works on radio SLAM are as follows: Ref. [31] (presenting a 2023 survey on radio SLAM), Ref. [32] (presenting in 2022 several radio SLAM filters for reduced receiver complexity), Ref. [33] (a very recent work published in 2025 on RIS-based SLAM), Ref. [34] (presenting an experimental validation of a radio SLAM full-processing chain on a wireless receiver in 2024), and Ref. [35] (proposing a robust snapshot-radio SLAM solution for mixed environments with Line of Sight (LOS) and Non-Line of Sight (NLOS) conditions in 2024). There are three main types of sensing and, thus, three main types of SLAM [29], listed as follows:

- *Monostatic SLAM*: When the transmitter and receiver are co-located on the same device (thus, they also share the same clock), the sensing part can rely on reflections on the surroundings sent by the device acting as a transmitter and captured by the same device, acting also as a receiver; the ‘sensed’ targets can be *disconnected*, meaning that they do not need to be equipped with a transmitter or a receiver themselves. A *connected* device refers to a device equipped with radio connectivity. Typically, with radio-based SLAM, monostatic SLAM is performed at the base-station side (which has more computational power than the User Equipment (UE)); the term monostatic SLAM did originate from robotics, wherein a robot performed monostatic SLAM based on vision signals [29].
- *Bistatic SLAM*: When there are two separate devices of interest, one acting as the transmitter (e.g., the base station) and the other one acting as the receiver (e.g., UE), receiving signals both from the LOS path (these control the part responsible for the positioning of the device) and from the NLOS path (i.e., reflections on the environment objects, which enable the sensing or location of those objects), single or multiple reflections on the environmental objects are possible.
- *Multistatic SLAM* is the most generic case, covering both of the previous cases as well, and it refers to situations involving multiple transmitters and receivers that transmit pilot signals for location and sensing. In bi- and multistatic configurations, the synchronization of time and frequency between the different devices, acting as transmitters and receivers, is not a given; thus, the SLAM algorithms should also take into account the clock and frequency inaccuracies.

The joint communication and positioning domain or JCAP has been seldomly addressed more in the existing literature so far, especially compared to the joint communication–sensing (ISAC/JSAC/ICAS/JCAS) and joint sensing–positioning domains (SLAM/JPAS). An example of a study addressing the JCAP concept can be found in [36], but JCAP is an abbreviation very rarely used nowadays, as many researchers address the joint positioning–communications aspects under the ISAC/JSAC/ICAS/JCAS umbrellas, based on the broader definition of sensing term, which can also include the positioning aspects.

In order to illustrate the current interest in the separate and the joint research areas encompassing the positioning, sensing, and/or communication research, an illustration based on a few simple keywords is depicted in Table 1. Only the most prevalent research areas are shown in the table, for the purpose of clarity (three separate and two joint research areas). The number of papers returned via keyword search is based on four selected databases: CORE (https://core.ac.uk/), Scopus (https://www.scopus.com), with institutional access, Google Scholar (https://scholar.google.com/), and a search of MDPI publishers (https://www.mdpi.com/search); the search was carried out in all fields (when available) or in the metadata (when a search involving all fields was not available). As seen in Table 1, even if the numbers across different databases vary significantly, they are all highly correlated and illustrate the breadth and width of various research domains (e.g., the radar and wireless communications domain are very broad research domains, while positioning and SLAM are much narrower research domains). Table 1 also illustrates that some keywords are more popular than others when referring to the same research areas (e.g., the term “Wireless positioning” is much more popular than the term “wireless navigation”, ISAC is preferred to JSAC as the terminology in use, the concept of radioSLAM is still very infrequently used in the research papers, etc.)

**Figure 2:**
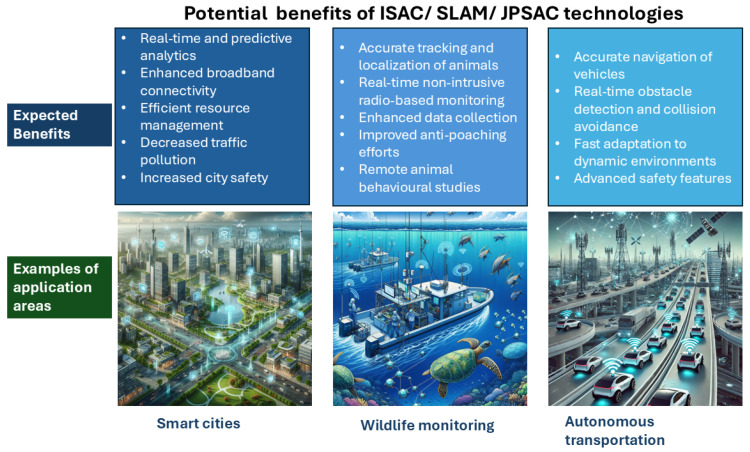
An illustration of the benefits of JPSAC solutions for various application areas (figure partially created with the Copilot Designer AI tool).

Figure 2 illustrates three potential application areas for JPSAC-related research and provides further details about how the SLAM, ISAC, or the more generic JPSAC algorithms can benefit the end users in various application areas.

The next section delves deeper in the details of what SLAM, ISAC, and JPSAC entail, what kind of methods are typically adopted, and which surveys and tutorials provide good overviews of these joint or integrated areas.

## 2. Brief State-of-the-Art Overview on Research Addressing Joint Radio-Based Communication, Sensing, and Positioning Aspects

The research on joint radio-based communications, sensing, and positioning is a fast evolving field, driven by the need to efficiently utilize the radio spectrum and to enhance the capabilities of modern wireless systems. The problems associated with JPSAC are typically complex optimization problems aiming to optimize the use of available resources such as bandwidth and power and to enable simultaneous data transmission, environmental sensing, and precise positioning. The main sub-research areas included under the wide JPSAC umbrella are:

- A waveform design to reach the desired JPSAC targets (see the discussion of typical performance metrics below) [28,37,38]
- Channel modeling and estimation under various scenarios (e.g., far-field and near-field, Multiple Inputs–Multiple Outputs (MIMO), massive Multiple Inputs–Multiple Outputs (mMIMO), and ultra-massive Multiple Inputs–Multiple Outputs (umMIMO), etc.) [25,29,34,36]
- Optimal resource allocation and management (e.g., beamforming strategies, power control, spectrum allocation, etc.) [15,39]
- Robust estimators and filtering for radio SLAM approaches [31,32,34,35,40]

The envisaged advantages of a ISAC, SLAM, or in general, of any JPSAC-related solution of integrating two or more of the three areas (communication, sensing, and positioning) are:

- An improved Spectral Efficiency (SE) compared to systems focusing on only one of the three areas (communication, sensing, and positioning);
- A reduction in device size, cost, and complexity with same quality of service or performance as the non-joint solutions;
- Decreased power consumption, especially at the mobile device side;
- Increased capacity or throughputs with lower Bit Error Rate (BER);
- Better use of existing resources (e.g., time, space, and frequency resources).

Some of the most relevant performance metrics in the joint processing of communication, sensing, and positioning are:

- Accuracy in terms of positioning and sensing, measured, for example, as Root Mean Square Error (RMSE) or Mean Absolute Error (MAE) between the estimate (e.g., position, velocity, orientation, etc.) and ground truth;
- Achievable data rates or channel capacity for a certain ISAC/JPSAC system;
- Ambiguity Function (AF)—this provides a two-dimensional representation of how a signal behaves in terms of time delays and Doppler frequency shifts; in a sensing context, it assesses the ability of a waveform to distinguish between targets at different ranges and velocities; in a positioning context, it provides the resolution and accuracy of the positioning system; and in a communication context, it helps in understanding the impact of time and frequency shifts on the signal;
- Cramer–Rao Lower Bound (CRLB)—this is a lower bound on the variance of any unbiased estimators, indicating the best possible accuracy that can be achieved for estimating a parameter given a certain amount of data; the parameter can be a sensing, a positioning, or a communication parameter;
- Detection probabilities in the context of positioning or sensing, measuring, for example, LOS/NLOS path detection, obstacle detection, etc.
- Data Information Rate (DIR)—this is the rate at which data can be transmitted while simultaneously performing sensing tasks; the higher the better;
- Mutual Information (MI)—this measures the amount of information that one random variable (e.g., a sensing-related parameter) contains about another random variable (e.g., a communication-related parameter);
- Signal to Interference plus Noise Ratio (SINR)—this measures the ratio of the signal power to the combined power of interference and noise, and is relevant in all three areas of positioning, sensing, and communications; the higher, the better;
- Squared Position Error Bound (SPEB)—this is a lower bound on the mean squared error of position estimates, indicating the best possible accuracy that can be achieved for the positioning part of the JPSAC systems.
- The sum rate—this represents the total data rate achieved by the system across all communication links and is a key performance indicator used to evaluate the efficiency and capacity of the communication aspect of the JPSAC systems.

The main methodological tools when dealing with joint performance metrics in two or three areas among communication, sensing, and positioning are listed in the fourth column of Table 2. This table summarizes the main surveys from the current literature on joint or integrated algorithms for receivers, simultaneously addressing at least two of the three above-mentioned targets (i.e., communication, positioning, and sensing) and relying on RF wireless signals. The fifth column in Table 2 also mentions the technologies addressed as examples in the listed surveys, with the note that most of the methods, algorithms, and performance metrics in these surveys are in fact applicable for a wide area of technologies and they are not limited only to the technologies mentioned in the table.

## 3. Emerging Topics in JPSAC Context

The emerging trends and topics in the ISAC, radio SLAM, and JPSAC contexts are discussed in the following subsections. Examples of open research questions are given in each subsection.

### 3.1. ELAA, XLMIMO, mMIMO, umMIMO, and LIS

ELAA, Extremely Large-scale Multiple Inputs–Multiple Outputs (XLMIMO), mMIMO, umMIMO, and Large Intelligent Surfaces (LIS) are different antenna categories that fall under at least one of these conditions: the antenna dimension is much larger than the wavelength (e.g., ELAA and LIS) or the number of antenna elements is massive (i.e., in the order of hundreds) or ultra-massive (thousands). The definitions of these terms are sometimes a bit ambiguous in the current literature with respect to their differences and similarities, though these terms are not exactly synonymous with each other.

The ELAA concept [47,48] refers to the deployment of a very large number of antenna elements over a large physical area. This can be achieved through distributed antenna systems or large-scale antenna arrays. ELAA can provide high spatial resolution and improved SNR by leveraging the large aperture, and this is particularly useful for applications requiring precise beamforming and high-resolution sensing.

A holographic MIMO antenna [29,49] uses a nearly continuous surface of reconfigurable and sub-wavelength-spaced antenna elements to create a holographic image of the electromagnetic field, allowing for the precise control of the wave fronts.

XLMIMO [50] refers to both systems with a massive number of antennas, often in the hundreds or thousands, thus covering the mMIMO and umMIMO categories, as well as to antennas with very large dimensions compared to the wavelength, thus covering the ELAA category.

The mMIMO and umMIMO [51] categories refer to antennas with a large and very large number of elements, though there is no unanimous agreement in the literature with respect to what represents a ‘large’ or a ‘very large’ number. A coarse rule of thumb is that antennas with few dozen to a few hundred elements fall into the mMIMO category, while antennas with more than a few hundred antenna elements fall into the umMIMO category.

The massive diversity and refined spatial resolution achieved by the ELAA, XLMIMO, mMIMO, umMIMO, and LIS antennas indicate a yet-to-be-harnessed potential for JPSAC. Examples of the research questions to be addressed in order to reach this potential include: how to overcome the beam-squirt or beam-squint effects (i.e., the unintentional change in the main beam direction during the beam-steering process, due to the frequency-dependent phases), and how to overcome the complexity and hardware challenges in the implementation of ISAC algorithms with ELAA/XLMIMO systems.

### 3.2. Low-Earth-Orbit Satellites

With the fast-pace deployment of satellites in LEO orbits, the research interest in the ISAC with LEO has also increased in the past few years. Some of the unsolved research questions in this area include how to manage the interference between multiple beams of LEO satellites serving different users, especially in densely populated areas, how to avoid congestion in the frequency bands shared by LEO satellites (e.g., particularly in the S-band, C-band, and millimeter-wave bands) when radar and communication waveforms co-exist in the same frequency bands, and how to increase the power and energy efficiency (as LEO satellites have limited resources) while, at the same time, ensuring fair resource allocation among users. A few of the recent research papers focusing on LEO ISAC are the works in [52] (addressing the problem of optimizing the energy efficiency) and in [53] (proposing a beam-squint-aware ISAC LEO technique).

### 3.3. Cell-Free or Distributed Networks

Cell-free or distributed networks refer to advanced network architectures where multiple distributed access points or base stations work together to provide seamless communication and sensing capabilities. In cell-free networks, there are no traditional cell boundaries. Instead, a large number of distributed access points jointly serve the users, providing uniform service quality across the coverage area. Typically, the access points are spatially distributed and work collaboratively to serve users and perform JPSAC tasks. Some of the unsolved research questions in this area are how to manage the interference between multiple distributed access points, how to efficiently allocate the resources (e.g., power, bandwidth, beams) among the distributed access points for maximum performance, and how to ensure precise time synchronization among distributed access points, when coherent signal processing is desired. The security in cell-free systems with ISAC functionalities has been recently addressed in [54]. The transmitter beamforming design for a cell-free mMIMO system with ISAC has been recently presented in [55]. A review of cell-free mMIMO concepts can be found in [56].

### 3.4. Near-Field Channel Modeling and Estimation

Near-field and far-field propagation are concepts in electromagnetics that describe how electromagnetic waves behave at different distances from a source, when distance is measured with respect to the signal wavelength and antenna dimension. Traditional communications, with a low-to-moderate number of antennas and wavelength in cm-Wave and mm-Wave frequencies reach the far-field mode very close to the emitter, and therefore the near-field effects can be ignored. However, with the advent of mMIMO and umMIMO and the interest in higher and higher carrier frequencies, moving towards the THz ranges, the near-field propagation mode can no longer be ignored and it has started to garner significant interest among researchers, especially over the past year [44,45,48,57,58,59].

According to [57], a signal is placed in near-field propagation conditions as long as its range to the emitter is below the near-field threshold $R_{NF}=\frac{2A^{2}}{\lambda }$ and it propagates according to far-field laws when its distance to the emitter becomes higher than $R_{NF}$, where *A* is the antenna’s maximum linear size (in m) and $\lambda =\frac{c}{f_{c}}$ is the signal wavelength (in m), with *c* being the speed of light (in m/s) and $f_{c}$ being the carrier frequency (in Hz). For example, a linear antenna with a moderate 8 antenna elements spaced at λ/2 apart and operating in the cm-Wave bands at 1 GHz would have a near-field threshold $R_{NF}=7.3$ m, which means that one could neglect the near-field propagation without a significant impact on the modeling, but an umMIMO antenna with 1024 antenna elements spaced at λ/2 apart and operating in the mm-Wave bands at 60 GHz would have $R_{NF}=2614.5$ m; thus, the near-field propagation mode can no longer be ignored.

Unlike the far-field situation, when the signal strength depends only on the distance between the transmitter and the receiver, but not on the angle, the signal strength in near-far conditions is dependent on both the angle and the distance.

The relevant open research questions here are, for example, how to derive unified channel models for far- and near-field propagation, how to address the increased complexity of the channel models in near-field conditions, and how to perform precise beam alignments.

### 3.5. Reconfigurable Intelligent Surfaces

The RIS or Intelligent Reconfigurable Surfaces (IRS) paradigm is also connected to some extent to the ELAA, XLMIMO, LIS, mMIMO, and umMIMO concepts addressed above. RIS is a more generic term than the above-mentioned antenna types and it refers to antennas which can dynamically control the propagation of the electromagnetic waves; this can be used to enhance the radio-based communications, sensing, and/or positioning tasks at the receivers by reflecting, refracting, or absorbing/blocking signals as to improve coverage, capacity, and energy efficiency. RIS has gained a lot of attention in the context of JPSAC in the past few years due to its unique potential to dynamically control the propagation environment. For example, such dynamical adjustments can improve the signal coverage and capacity, especially in challenging environments such as urban areas with many obstacles, and they can help in managing interference and in redirecting signals on additional NLOS paths when LOS is blocked. A comprehensive survey on RIS in ISAC can be found in [60]. Some of the unsolved research questions pertaining to RIS applicability in JPSAC context are: how to best model the reflection losses and the specular (i.e., similar to a mirror’s reflections, with the incident angle equal to the reflected angle) and non-specular reflections on RIS, how to distinguish the signals coming from different emitters through multiple bounces or reflections on RIS, and how to build realistic RIS channel models and perform accurate channel estimations.

### 3.6. Machine Learning/Artificial Intelligence

The ML and AI paradigms have also permeated into the JPSAC context. This is a very broad and emerging area and it has many open challenges, both ML specific (e.g., explainability, reproducibility, fairness, data scarcity, and accessibility) and JPSAC-specific (scalability, real-time processing, access to ground truth for supervised-data labeling, security and privacy, computational constraints especially on energy-constrained devices, etc.).

Examples of recent works discussing or applying ML/AI in the context of JPSAC are: Ref. [9] (proposing a deep reinforcement learning method for RIS-based ISAC), Ref. [61] (a magazine survey of AI uses in the context of ISAC), Ref. [62] (ML for interference management in the context of ISAC context), and [63] (a neural-network-based estimation of angles in bistatic ISAC).

### 3.7. Polarization Aspects

In the context of radio signals, polarization refers to the orientation of the electric-field component with respect to the magnetic-field component of the electromagnetic wave as it propagates through space. It can be, for example, linear (i.e., electric and magnetic fields are perpendicular to each other and each of them oscillates in a single plane), circular (both electric and magnetic fields rotate in a circular motion with respect to the wave propagation direction, and they are perpendicular to each other), or elliptical (similar to the circular orientation, but with elliptical motions instead of circular ones). Traditionally, the effects of polarization have been ignored in the context of positioning and sensing, which has also frequently been the case in the context of wireless communication research. However, polarization can affect resolution and accuracy in sensing and positioning, and mismatched polarizations can lead to significant signal loss and reduced communication efficiency. For example, a proper alignment of polarization between the transmitter and receiver can reduce interference and improve the SNR; also, using different polarizations on targets (i.e., polarization diversity) can enhance target detection and resolution. The potential benefits of integrating the polarization dimension into localization applications has recently been studied in [64]. The open research questions include how to avoid ambiguities that arise when the nuisance parameters (i.e., those parameters not relevant in the JPSAC solution) are jointly modeled or searched for in a combined manner, how to deal with challenging NLOS and 3D scenarios that increase the estimation complexity, how to deal with the complexity of polarimetric data, and how to carry out an accurate calibration of polarimetric sensors.

### 3.8. Snapshot-Radio SLAM

The radio SLAM or radio-based SLAM refers to solutions for the joint estimation of various positioning/sensing parameters (e.g., position, velocity, orientation, etc.) of both the target devices and surroundings. Notable surveys about radio SLAM can be found in [29,31]. Both radio SLAM and snapshot-radio SLAM are currently regarded as complex and challenging research issues. Snapshot-radio SLAM refers to estimators relying on a single snapshot of radio measurements, rather than relying on continuous data collection; this can serve to decrease the power consumption, especially at the side of the mobile device, as well as offer fast real-time positioning and sensing estimates. A basic radio SLAM problem includes at least 7 parameters to be estimated, assuming a single reflection point on a surrounding object, 3D estimates, and desynchronized clocks at the transmitter and receiver; these 7 parameters are the x, y, z coordinates of the mobile target, the x, y, z coordinates of the scatterer or reflector, and the unknown clock bias between the transmitter and receiver clocks. If we assume multiple reflection points (e.g., on RIS surfaces) and also introduce, as unknowns, the device and surrounding objects’ rotations (i.e., pitch, roll and yaw in 3D space), as well as the velocities and the wave polarization, one can easily see that the complexity of the estimator increases tremendously. In addition, since snapshot-radio SLAM relies on a single snapshot of data, it has to extract as much information as possible from limited measurements, and this raises issues of robustness under fast-changing environments and limited data. A few of the recent works addressing snapshot-radio SLAM can be found in [34,35,40,65].

### 3.9. Terrestrial Networks (TN) and Non Terrestrial Networks (NTN) Integration

Traditionally, up to 5–10 years ago, the research focusing on TN was separated from the research focusing on NTN (e.g., satellite signals and systems). However, similarly to the convergence of the communication, sensing, and positioning research areas, a convergence betweenTN and NTN research has happened in the past few years, which has also been reflected in the standardization committees and published documents (e.g., the 3GPP TR 38.821 document details this integration).

This integration of TN and NTN in the context of ISAC is discussed, for example, in [66]. The seamless localization and communication achievable based on a TN-NTN integration is discussed in [67]. Jammer detection in JSAC with integrated TN-NTN networks is discussed in [68]. Nevertheless, many research questions still remain unanswered in the area of integrated TN-NTN networks, such as: how to ensure seamless interoperability between the TN and NTN components, how to perform optimal data fusion with heterogeneous TN and NTN signals, how to overcome the long propagation delays in NTN signals in order to achieve low-latency JPSAC solutions, how to allocate the TN and NTN resources in a dynamic way in order to adapt to varying network conditions and user demands, etc.

### 3.10. Waveform Design

The research field of waveform design in its broader sense is not, strictly speaking, an emerging one, as researchers have focused their attention on waveform design in various wireless areas for decades. However, the novelty of the waveform design in the context of ISAC stems from the dichotomy between either designing—and then embedding—two simultaneous signal waveforms, one for communication and one for sensing targets, or designing a single waveform with dual functionality. The former approach is also known as RCC, while the latter is referred to as DFRC [69]. A few of the main open research questions relevant to this area involve determining which approach is more suitable for the desired application, how to optimally design such waveforms, and which are the most suitable optimization metrics and optimization-solving approaches. In the case of RCC, there will be interference between the two simultaneous waveforms, and this must be taken into account. Index Modulation (IM) is one recently proposed method for DFRC modulation design, in which the information-bearing signal (i.e., the communication waveform) is indexed or embedded in the radar waveform. IM can be carried out in the frequency, antenna, phase, or space domains as well as in a hybrid domain involving all four [69]. Typically, hybrid approaches can achieve a lower complexity than standalone ones, while spatial-path approaches achieve the best communication rates [69].

### 3.11. Standardization Efforts

Among the multiple JPSAC domains (see Figure 1), the research in the ISAC area has garnered the most attention from the standardization committees. For example, European Telecommunications Standards Institute (ETSI) has created an Industry Specification Group (ISG) for ISAC in November 2023, and this group is working actively to define use cases, develop advanced channel models, and propose Key Performance Indicators (KPIs) and evaluation methodologies for ISAC. ISAC standardization efforts can also be observed at the 3GPP level, with recent documents addressing near-field channel models (e.g., 3GPP TR 38.901 and 3GPP TR 38.900 documents) or the integration of TN with NTN models (e.g., 3GPP TR 38.821 document). A description of the latest progress in 3GPP regarding ISAC channel modeling can be found in [70].

## 4. Ongoing Horizon Europe Projects Related to JPSAC

Related to the emerging topics in JPSAC areas, we detail the ongoing research projects in Europe. In particular, the JPSAC-related research areas have garnered a particularly high level of interest in European research, and many projects, especially MSCA training networks, are working to address the challenges of ISAC and radio SLAM. We have summarized the ongoing European research in Table 3. All projects listed in Table 3 are ongoing Horizon Europe projects as of January 2025. It can be easily seen from this table that the key research areas listed in Table 3 correspond to most of the emerging topics discussed in Section 3. The international landscape of research and development projects is likely to resemble research developments in Europe to a significant degree, but we have not detailed it here due to a lack of user-friendly country-agnostic search interfaces for research projects at an international level. As an illustrative and partial comparison of EU JPSAC-related research with the research recently funded by the United States National Science Foundation (NSF) (https://www.nsf.gov/awardsearch/), recent NSF themes include: learning-assisted integrated sensing, communication, and security for 6G Unmanned Aerial Vehicles (UAV) networks, dedicated waveform synthesis and performance assessment for ISAC, and AI-assisted waveform and beamforming design for ISAC.

## 5. Conclusions

Communications, sensing, and positioning tasks in emerging wireless transceivers can no longer be seen as separate tasks, as they have been converging over the past few years, meaning that they need to be optimized in a joint or integrated manner in order to achieve the ambitious targets set for future wireless communications (e.g., 6G and beyond). The convergence of positioning, sensing, and communication tasks based on radio signals has been referred to in this editorial under the terminology encompassing JPSAC, attempting to unify all the different notations from the literature in this field; this has been illustrated through a comprehensive Venn diagram, in order to point out the different research directions under the JPSAC umbrella. Within the broader JPSAC terminology, the terms ISAC and radio SLAM are the most frequent, yet, at the time of writing this editorial, there is no universally adopted terminology in this research area, and many similar terms, such as JCAS, JSAC, DFRC, RCC, etc., are also widely used. The main purpose of JPSAC-related research is to ensure optimal use of the available radio resources, such as the bandwidth, power, or carrier frequencies. Our editorial summarized nine of the existing comprehensive surveys in JPSAC-related areas (see Table 2) and provided a top-level overview of the typical research areas and typical KPIs under the JPSAC umbrella. At its core, this editorial focused on emphasizing 11 research directions in JPSAC-related areas and pointing out several open research questions and research challenges in this field. A brief overview of the European landscape of ongoing research projects funded by the Europe Horizon program was also provided, in order to show that these projects also coincide with the emerging topics in JPSAC areas.

JPSAC-related research is at the forefront of 6G technology development, enabling high-precision sensing and communication and complete coverage of the positioning, sensing, and communications solutions, which are essential for advanced applications such as autonomous vehicles, smart cities, or industrial automation. Advancing the research in these JPSAC-related areas contributes to the broader scientific understanding of wireless communication and sensing, as it helps to address fundamental challenges and pushes the boundaries of what is possible with current technology.

Some of the expected benefits to society at large, to be obtained by integrating radio-based positioning, communication, and sensing tasks in future wireless receivers, are enhanced energy and spectral efficiency; better resource utilization, leading to the more efficient use of the radio spectrum and hardware resources; improved safety and security, such as Advanced Driver Assistance Systems (ADAS) in vehicles, intrusion detection, and environmental monitoring; enabling innovative applications, such as smart cities, where sensors monitor traffic, air quality, and energy usage; e-health applications, where integrated solutions can offer remote patient monitoring and fall detection; increased economic growth by creating new business opportunities and markets; and, in general, contributing to a more connected, efficient, and safer society.

## Figures and Tables

**Table 1:** Examples of the number of citations in five selected research areas related to positioning, sensing, and communications (as of 10 January 2025).

Area	Search Keywords	CORE	Scopus	Google Scholar	MDPI
Positioning	“wireless positioning”	1153	3360	9000	58
	“radio localization”	662	1789	3600	24
	“wireless navigation”	119	189	906	5
Sensing	“wireless sensing”	5357	16,038	45,100	255
	“radar”	507,727	789,674	4,780,000	11,956
Communications	“wireless communications”	114,657	830,169	1,160,000	3196
	“radio communications”	28,624	139,957	287,000	226
	“telecommunications”	446,714	1,586,347	3,500,000	10,093
ISAC	“JSAC”	9498	16,279	59,700	2
	“ISAC”	24,192	11,354	172,000	441
SLAM	“SLAM”	120,114	64,035	738,000	1213
	“radio SLAM”	77	199	329	1

**Table 2:** The top surveys in the current literature addressing joint radio-based positioning, sensing, and communication notions (e.g., ISAC, SLAM, JPSAC, etc.).

Ref.	Year	Brief Description	Methods	Wireless Technologies	Metrics
[26]	2021	An overview of signal-processing techniques and signal and channel mathematical models for ISAC/JCR; a comparison of radar and communication goals	Three design methodologies: communication-centric, sensing-centric, and joint design, each with own performance metrics; multi-beam optimization; time–frequency domain-based optimization; etc.	802.11ad	Communication: capacity, SE, BER; Sensing: detection probability CRLB, MI, AF
[41]	2022	A survey on mm-Wave indoor localization and sensing, including relevant channel models	Both geometric and ML algorithms are addressed and compared, using time, angle, or power measurements; a variety of analytical tools are overviewed	5G/6G	Location accuracy, LOS and obstacle detection probability
[42]	2023	A survey on ISAC signal design, processing, optimization, and future trends in ISAC	Use of OFDM with other methods such as LFM and phase coding and of OTFS for the waveform design; frequency-based and super-resolution methods for the signal-processing part; various adaptive and non-adaptive optimization methods	5G/6G	Target resolution, AF, Doppler sensitivity and tolerance, PAPR, MI, DIR, complexity, accuracy, CRLB, …
[43]	2023	A survey of ICAS in the context of MIMO	Detailed discussions on various precoding and waveform design mechanisms and various optimization mechanisms	5G/6G	User rate, capacity, detection probability, estimation resolution, …
[29]	2024	A very comprehensive survey and tutorial on ISAC and radio SLAM and on how signal processing, optimization, and ML can be leveraged in 6G context; ISAC with large apertures and RIS	Detailed mathematical models; 3GPP standardization overview; a wide overview of methodologies for waveform and codebook design and channel estimation in ISAC; ML-based solutions for radio SLAM; definitions of snapshot, filtering, and smoothing approaches, etc.	5G/6G	Accuracy measured in terms of positioning errors, target detection probabilities, SINR, positioning and sensing ranges, sum rate, SPEB ,…
[44]	2024	A survey on near-field ISAC and of the integration of ELAA with ISAC; channel models for near-field and far-field scenarios; a brief discussion on standardization efforts	Three addressed cases: sensing-centric, communication-centric, and joint communication and sensing via a sum-weighted approach; the integration of ISAC with NOMA; the exploitation of spatial resolution and advanced beamforming of near-field	5G/6G	SNR, beam-pattern gain, sum rate
[45]	2024	A magazine surveying ISAC in THz bands; a comparison with mm-Wave bands is also provided	Challenges in THz bands, such as beam-split or beam-quint effects, near-field effects, distance-dependent bandwidths, and the broadening of the absorption lines; umMIMO and RIS are proposed as solutions to counter the effects of the huge path losses	6G	SE, power consumption, SNR
[38]	2024	A survey on waveform design methods in ISAC	Both single-waveform and dual-waveform (DFRC/(RCC) designs are outlined by defining various optimization problems in terms of the objective function and constraints	mostly OFDM-based signals	RMSE, detection probability, SINR, radar estimation rate and communication rate ,…
[46]	2024	A survey on the design of RF front-ends for ISAC applications	Focus on the reconfigurability of frequency, gain, bandwidth and linearity of the LNA and front-end mixers	5G	noise figure, 1 dB compression point, third-order intercept point and other front-end-related metrics

**Table 3:** Ongoing Horizon Europe projects addressing various aspects in JPSAC areas.

Project	Key Research Areas Related to JPSAC
6G-DISAC (https://www.6gdisac-project.eu/)	Distributed intelligent sensing and communication; ISAC for 6G
6th sense(https://dn6sense.eu/)	Sensing-assisted communications, communications-assisted sensing, and multi-functional AI-JCAS
INSTINCT (https://cordis.europa.eu/project/id/101139161) (accessed on 8 December 2023)	Distributed sensing and communications; ICAS for 6G
ISAC-NEWTON (https://cordis.europa.eu/project/id/101169496) (accessed on 5 July 2024)	ISAC for perceptive mobile networks in 6G; enhanced positioning and localization
iSEE-6G (https://isee6g.eu/)	Joint communication, computation, sensing, and power transfer; RIS and agile beamforming; cross-layer designs and system-level solutions for 6G
ISLANDS (https://www.islands-mscadoctoralnetwork.eu/)	Automotive radar-centric ISAC system design; radio-based SLAM techniques; resource allocation for ISAC-enabled vehicular networks
MiFuture (https://mifuture.tsc.uc3m.es/)	Waveform design for ISAC; mm-Wave positioning and sensing; joint positioning and spatial resource allocation for cell-free systems; ISAC for mMIMO and umMIMO systems
MultiX (https://cordis.europa.eu/project/id/101192521/) (accessed on 7 November 2024)	Multi-sensor, multi-band, multi-static ISAC solutions in 6G; fully flexible ISAC deployment in 6G; addressing mobility challenges for sensing and localization services
RIXISAC (https://cordis.europa.eu/project/id/101155506/) (accessed on 28 March 2024)	Design of RIS for ISAC; AI-driven resource allocation and network optimization
SMARTTEST (https://dn-smarttest.eu/)	Design of ISAC devices and algorithms for contact-free, continuous, and proactive remote health monitoring

## Abbreviations

The following abbreviations are used in this manuscript:

**3GPP**	Third-Generation Partnership Project
**4G**	Fourth generation of cellular networks
**5G**	Fifth generation of cellular networks
**6G**	Sixth generation of cellular networks
**ADAS**	Advanced Driver Assistance Systems
**AF**	Ambiguity Function
**AI**	Artificial Intelligence
**BER**	Bit Error Rate
**CRLB**	Cramer–Rao Lower Bound
**DFRC**	Dual-Functional Radar Communications
**DIR**	Data Information Rate
**ELAA**	Extremely Large Aperture Arrays
**ETSI**	European Telecommunications Standards Institute
**GNSS**	Global Navigation Satellite Systems
**IoT**	Internet of Things
**ICAS**	Integrated Communications and Sensing
**IM**	Index Modulation
**IRS**	Intelligent Reconfigurable Surfaces
**ISAC**	Integrated Sensing and Communications
**ISG**	Industry Specification Group
**JCAP**	Joint Communications and Positioning
**JCAS**	Joint Communications and Sensing
**JCR**	Joint Communications and Radar sensing
**JPAS**	Joint Positioning and Sensing
**JPSAC**	Joint positioning, sensing, and communications
**JSAC**	Joint Sensing and Communications
**KPIs**	Key Performance Indicators
**LEO**	Low Earth Orbit
**LFM**	Linear Frequency Modulation
**LIS**	Large Intelligent Surfaces
**LNA**	Low Noise Amplifier
**LOS**	Line of Sight
**MAE**	Mean Absolute Error
**MI**	Mutual Information
**ML**	Machine Learning
**MIMO**	Multiple Inputs–Multiple Outputs
**mMIMO**	massive Multiple Inputs–Multiple Outputs
**NTN**	Non Terrestrial Networks
**NLOS**	Non-Line of Sight
**NOMA**	Non-Orthogonal Multiple Access
**NSF**	National Science Foundation
**OFDM**	Orthogonal Frequency Division Multiplexing
**OTFS**	Orthogonal Time Frequency Space
**PAPR**	Peak to the Average Power Ratio
**PNT**	Positioning, navigation, and Timing
**RCC**	Radar Communications Coexistence
**RF**	Radio Frequency
**RIS**	Reconfigurable Intelligent Surfaces
**RMSE**	Root Mean Square Error
**SE**	Spectral Efficiency
**SINR**	Signal to Interference plus Noise Ratio
**SLAM**	Simultaneous Localization and Mapping
**SNR**	Signal to Noise Ratio
**SPEB**	Squared Position Error Bound
**TN**	Terrestrial Networks
**UAV**	Unmanned Aerial Vehicles
**UE**	User Equipment
**umMIMO**	ultra-massive Multiple Inputs–Multiple Outputs
**XLMIMO**	Extremely Large-scale Multiple Inputs–Multiple Outputs
